# Supplementary figures and images for: Chronic β3‐AR stimulation activates distinct thermogenic mechanisms in brown and white adipose tissue and improves systemic metabolism in aged mice
Source: Aging Cell. 2024 Aug 23;23(12):e14321. doi: 10.1111/acel.14321 (PMC11634714; doi:10.1111/acel.14321)

Suppl Fig. 1

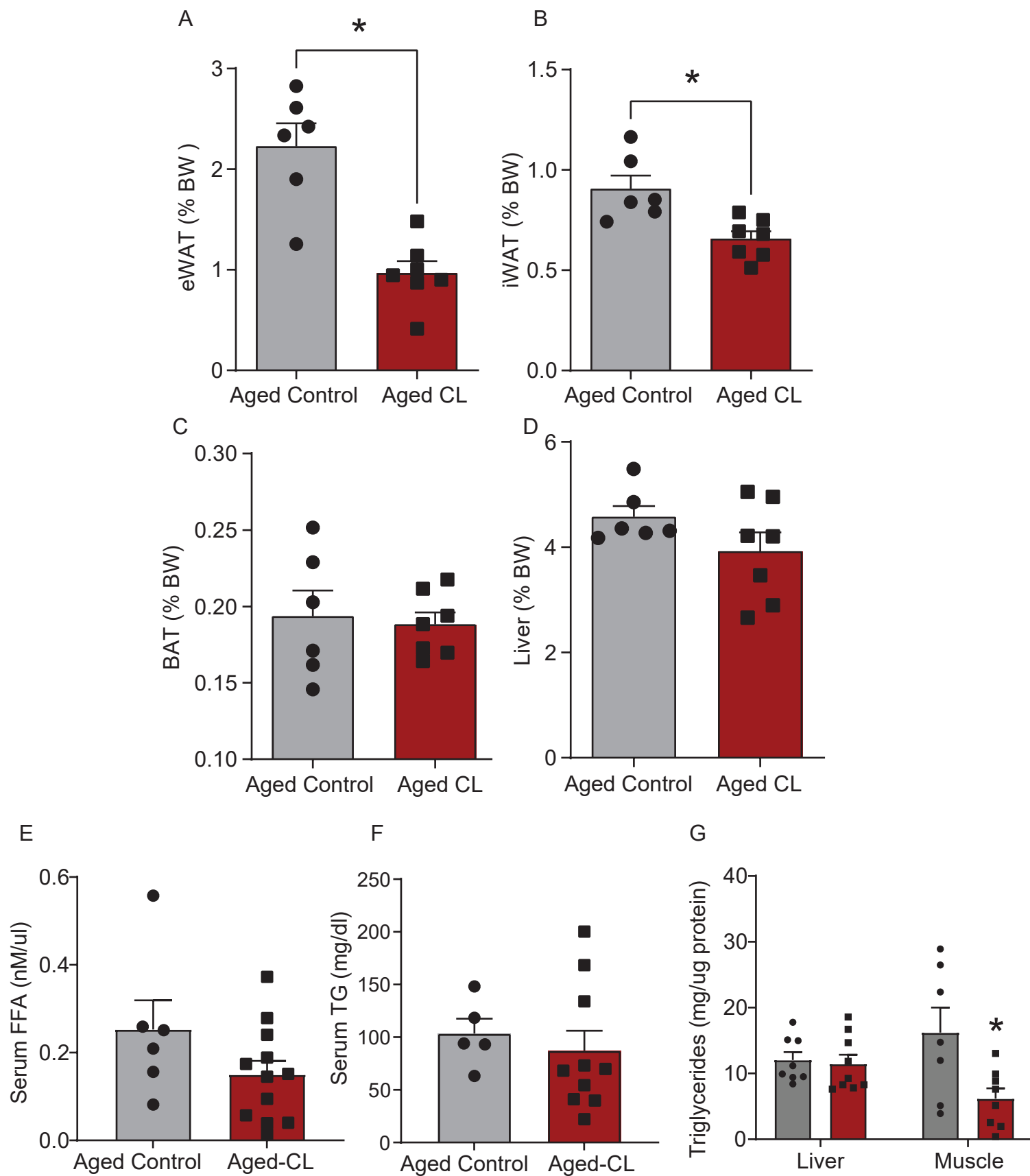

Suppl Fig. 2

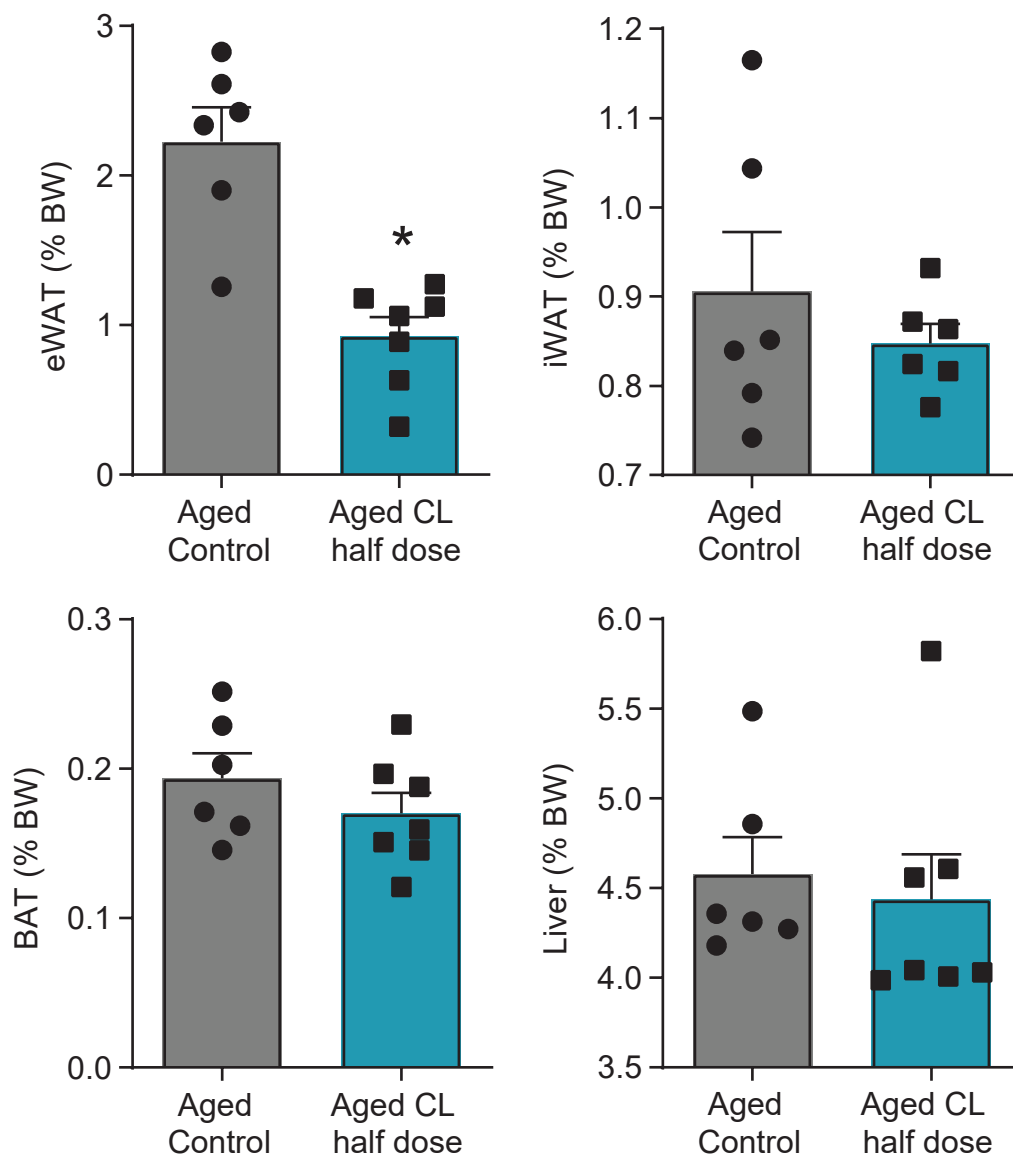

Supplement: Supplementary file 1 — Data S1. [file ACEL-23-e14321-s001.pdf]
